# Oxidizing and Nano-dispersing the Natural Silk Fibers

**DOI:** 10.1186/s11671-019-3080-1

**Published:** 2019-07-25

**Authors:** Ke Zheng, Yanlei Hu, Wenwen Zhang, Juan Yu, Shengjie Ling, Yimin Fan

**Affiliations:** 1 Jiangsu Co-Innovation Center of Efficient Processing and Utilization of Forest Resources, Jiangsu Key Lab of Biomass-based Green Fuel & Chemicals, Key Laboratory of Forestry Genetics & Biotechnology (Nanjing Forestry University) of Ministry of Education, College of Chemical Engineering, Nanjing Forestry University, Nanjing, 210037 China; 2 School of Physical Science and Technology, ShanghaiTech University, Shanghai, 201210 China

**Keywords:** Silk, Oxidation, Negatively charged nanofibers, Aggregating-redispersing

## Abstract

Natural *Bombyx mori* silk (BS) and *Antheraea pernyi* silk (AS) were oxidized in sodium hypochlorite (NaClO) solutions. Thereafter, individual silk nanofibers (SNs) were achieved after sonicating the oxidized silk slurries, where the diameters of the resultant SNs were ~ 100 nm and several micrometers in length. Thin membranes were formed by casting the SNs, which had optically transparent (above 75% transmission), mechanically robust (~4.5 GPa of Young’s modulus), and enhanced wetting properties. An interesting aggregating-dispersing (re-dispersing) process by using these SNs was strongly regulated by adjusting the pH values. Consequently, the negatively charged SNs could be concentrated up to ~ 20 wt% (100 times that of the initial dispersion) and offered extraordinary benefits for storage, transportation, and engineering applications.

**Electronic supplementary material:**

The online version of this article (10.1186/s11671-019-3080-1) contains supplementary material, which is available to authorized users.

## Introduction

Materials with hierarchical structures are omnipresent in natural biological systems [1, 2]. They provide a diversity of functions due to the primary properties of the polymers and functional adaptation of the structures at each hierarchy [3–5]. To engineer artificial materials with enhanced functions that reproduce such special properties, extraction processes that retain the original nanostructures of the polymers have been desired [6–10]. A variety of studies have been devoted to isolating polysaccharide nanofibers (e.g., cellulose and chitin) from their fiber composite structures using chemical, physical, and biological approaches [11–13]. In particular, completely individualized and highly crystalline nanofibers have been obtained by employing the 2,2,6,6-tetramethylpiperidine-1-oxyl radical (TEMPO)-mediated oxidation of native cellulose/chitin, followed by mild mechanical treatment [14, 15]. However, economic and environmental issues still remain; these existing techniques for nanofibril isolation require expensive and/or toxic reagents, such as TEMPO and hexafluoroisopropanol (HFIP). More importantly, the low concentration of resultant nanofibers dispersion limited their storing, transporting, and applications.

Animal silks spun by a wide range of insects and spiders also possess hierarchical fibrous structures [16, 17]. These protein molecules are in the form of assembled fibrils from the nanoscale to the macroscale, resulting in outstanding mechanical and biochemical properties in the silk materials [18–21]. To achieve silk nanostructures, however, extraction processes remain challenges due to (i) the complex hierarchical structure, (ii) high crystallinity, and (iii) the adhesion between micro-/nano-fibrils of silk fibers. Ultrasonic treatment has been applied to split silk fibers [22]; however, the resultant nanofibers were intertwined and lacked processability. The partial dissolution of silk fibers using salt-formic acid system presented unstable tree-like nanofiber bundles [23]. An integrated approach using partial dissolution and ultrasonication resulted in silk fibers that were downsized to the diameter of a single nanofibril [24], while the aspect ratio and yield of such nanofibers has yet to be improved.

To address these issues, we have elaborated an easy and scalable strategy to extract full-sized mesosilks [25]. Similar to the isolation of polysaccharides [26], carboxyl groups were introduced onto *Bombyx mori* silk (BS) and *Antheraea pernyi* silk (AS) fibers for nanofibers dispersing via electrostatic repulsion; however, the redundant chemicals, such as TEMPO and sodium bromide (NaBr), were excluded because selective oxidation was unnecessary. Herein, we disclosed the effectiveness of this process for yielding individual nanofibers with high aspect ratios. Optically transparent, mechanically robust and enhanced wetting properties were obtained in the resultant silk nanofiber (SN) membranes. In comparison with those polysaccharides-based nanofibers (i.e., cellulose and chitin nanofibers), interesting aggregating-redispersing properties of the SNs were regulated by pH values.

## Materials and Methods

### Oxidation of the Disassembled Silk Fibers

The disassembled silk fibers were prepared from raw *Bombyx mori* (or *Antheraea pernyi*) silkworm fibers (Xiehe Silk Co., China). Briefly, 5 g of the silk fibers were boiled for 30 min in an aqueous solution of 0.02 M sodium carbonate with a weight ratio of 1:400, followed by thorough washing in distilled water and then air drying. Then, the degummed silk fibers were immersed in formic acid (88 wt%) solution with a weight ratio of 1:20. The mixture was incubated at room temperature for at least 1 h and then homogenized at 10,000 r/min for 3 min to obtain a suspension. The disassembled silk fibers were obtained in a solid state after centrifuging the suspension at 8000 r/min.

For the oxidation, disassembled silk fibers were washed to pH 7 and cut into short pieces that were several centimeters long, and a desired amount of sodium hypochlorite (NaClO) solution was added into 100 ml of water with 1 g of the disassembled silk fibers. Sodium hydroxide (NaOH) was continuously added into the mixture to maintain the pH at 10. When NaOH consumption was no longer observed, the reaction was quenched by adding drops of 0.5 M hydrochloric acid (HCl) to adjust the pH to 7. Then, the water-insoluble fraction was centrifuged at 10000 r/min and washed several times. Finally, silk nanofibers were obtained after treating the water-insoluble fraction with an ultrasonic homogenizer at 19.5 kHz with a 300 W output power for 20 min. An ice-water bath was employed to avoid the overheating during the long time of ultrasonication.

### X-ray Diffraction Analysis of the Oxidized Silk Fibers

The X-ray diffraction (XRD) experiments were performed using an Ultima IV multipurpose X-ray diffraction system (Ultima IV, Rigaku, Japan) with a Cu-Kα source (*λ* = 0.1542 nm). The voltage and current of the X-ray source were 40 kV and 30 mA, respectively. The deconvolution results of the oxidized silk fibers were analyzed using PeakFit software (4.0). The numbers and positions of the peaks were defined from the results of the second derivatives from the spectra and fixed during the deconvolution process. The bandwidth was automatically adjusted by the software.

### Morphology Observations of the Nanofibers

To observe the formation of the various nanofibers, the dispersion was diluted to 0.01 wt%. For the scanning electron microscopy, a 10 μL aliquot of the diluted dispersion was placed on a silicon wafer and then air dried. The samples were coated with gold and palladium and imaged using a JEOL-JSM 7600F (JEOL, Japan) SEM at a voltage of 5 kV. For the transmission electron microscopy (TEM), a 10-μL aliquot of the diluted dispersion was placed on a carbon-coated Cu electron microscopy grid. The excess liquid was absorbed by filter paper then air-dried. The sample grid was observed at 80 kV using a Titan 80-300 (FEI, U.S.) transmission electron microscope. The sizes of the nanofibrils were analyzed with ImageJ software (1.48) developed at the National Institutes of Health in the USA.

### Mechanical Testing

The BS, AS, CN (cellulose nanofiber), and ChN (chitin nanofiber) membranes with a thickness of approximately 50 μm were cast by using a solvent evaporation method. Each nanofiber membrane was tailored into several strips with lengths of 60–80 mm and diameters of 5 mm, and they were stretched by an electronic universal testing machine (AG-Xplus, SHIMADZU, Japan) to determine their mechanical properties. In this test, the initial interval of the fixtures was 20 mm, and the stretch speed was 1 mm/min.

### Optical and Wetting Properties

Light transmittances of the various membranes with a thickness of 25 μm were determined from 350 to 800 nm using an Ultrospec 2100 pro spectrometer from Amersham Biosciences.

A drop meter (Kyowa Interface Science Co., Ltd.) was employed for the contact angle measurements. Image analyses were performed automatically from the shapes of 4 μL distilled water droplets dropped onto the membranes within ~ 0.5 s.

## Results and Discussion

### Oxidation and Isolation of Silk Nanofibers

**Fig. 1:**
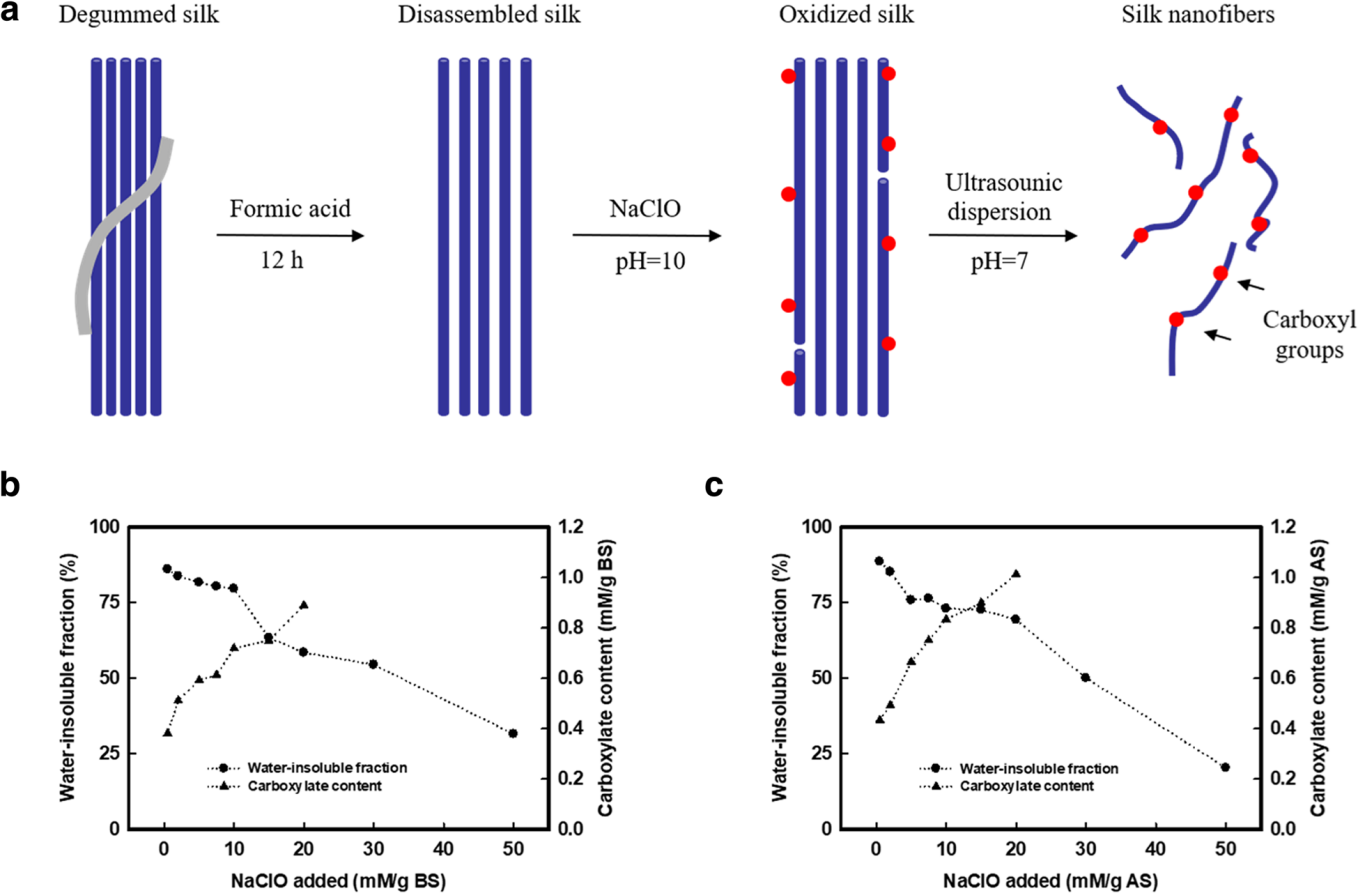
Process diagram of SNs and carboxyl content of BS and AS. **a** Schematic of oxidation and dispersing of silk fiber to silk nanofibers (SNs). **b** The content of carboxyl groups and weight remaining of water-insoluble fraction after oxidation of *Bombyx mori* (BS) corresponding to the addition of sodium hypochlorite (NaClO). The carboxyl content increased from 0.293 to 0.889 mM/g BS (NaClO addition was 20 mM/g protein) with 58.52 wt% of protein remaining. **c** For *Antheraea pernyi* silk (AS). The carboxyl content increased from 0.347 to 1.013 mM/g AS (NaClO addition was 20 mM/g protein) with 69.30 wt% of protein remaining

Figure 1a presents the strategy for isolating nanofibers from silk fiber materials. We first employed a pretreatment process to disassemble these silk fibers by treating with formic acid (no chemical reaction was occurred between amino acid or hydroxyl groups with formic acid as shown in Raman spectra in Additional file 1: Fig. S1 and the relevant discussion of Additional file). This pretreatment disassembled the silk fibers to microfiber structures with widths of 5–20 μm (Fig. 1a). Then, sodium hypochlorite (NaClO) was employed to oxidize/partially dissolve (degrade) the disassembled silk fibers. Sodium hydroxide (NaOH) was continuously added into the mixture to maintain the pH at 10, according to the conditions for the TEMPO (2, 2, 6, 6-tetramethylpiperidine-1-oxy-radical)-mediated oxidation of polysaccharides by using TEMPO/NaClO/NaBr system, while, in this case, the TEMPO and NaBr were no longer needed for the oxidation of silk fibers due to the limited reactive amino acids in silk fibroin sequences. The initial silk fibers had a carboxyl concentration of ~ 0.3 mM/g of protein, which was attributed to the aspartic and glutamic acids in the molecular sequence [27]. Thereafter, the carboxyl content of the oxidized silk increased approximately linearly following the addition of NaClO, due to the oxidation of hydroxymethyl groups on the serine residues. When the NaClO addition reached 20 mM/g of protein, the final carboxyl concentration of the oxidized silk was 0.889 and 1.013 mM/g of protein for BS and AS, respectively (Fig. 1b, c). However, excess amounts of NaClO may have degraded the silk fibers [28]. For example, the water-insoluble fraction of BS and AS was 58.52 and 69.30 wt% respectively, at the NaClO addition of 20 mM/g of protein. The weight loss of the water-insoluble fraction after oxidation suggested that the NaClO addition of ≤ 10 mM/g of protein was acceptable (over 75% of the protein remained), with respect to limited degradation during the oxidation. Therefore, we employed 10 mM of NaClO per gram of protein to oxidize BS and AS fibers, where the carboxylate content is 0.724 and 0.837 mg/g of protein for BS and AS, respectively.

**Fig. 2:**
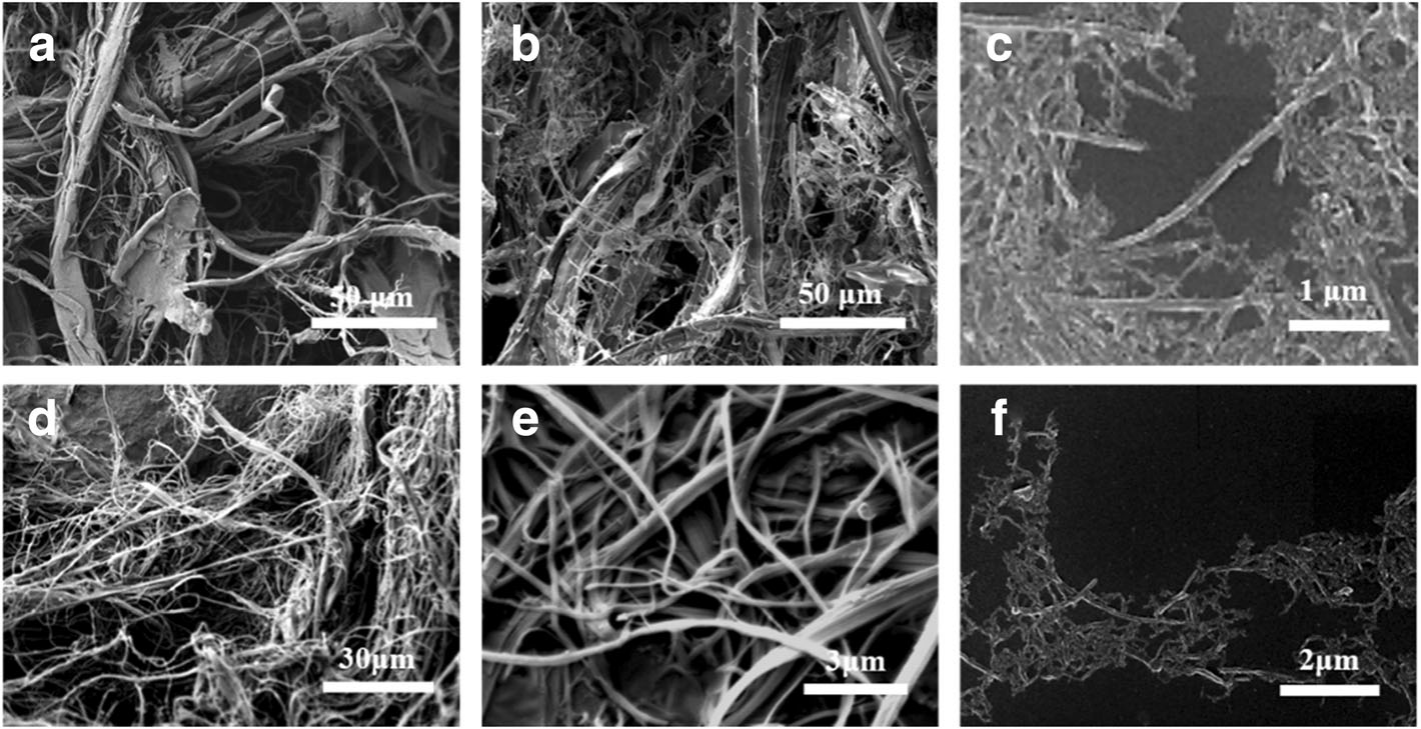
Representative SEM observation of resultant silk fibers in each process. **a** Disassembled BS fibers after formic acid pretreatment, **b** oxidized BS fibers, and **c** the BS nanofibers with a diameter of 105 ± 27 nm. **d** Disassembled AS fibers after formic acid pretreatment, **e** oxidized AS fibers, and **f** the AS nanofibers with a diameter of 112 ± 33 nm. The contour length of BS and AS nanofibers is more than 1 μm

Finally, nanofibers were achieved after treating the water-insoluble fraction with an ultrasonic homogenizer (Fig. 2). The scanning electron microscopy observations revealed that the oxidation loosened the silk at the microlevel, forming fibers with diameters of several microns, and the sonication treatment further dispersed them into nanofibers with a diameter of 105 ± 27 nm (Fig. 2c). Compared to other processes [24], which mostly exfoliate the surface layer of the silk fibers, a final yield of ~ 50% based on the oxidized silks was obtained for the nanofibers due to the electrostatic repulsive forces in the oxidized silks. A similar strategy was applied to AS fibers as well. The diameter of the resultant AS nanofibers was 112 ± 33 nm, and the contour length was more than 1 μm (Fig. 2f).

### The Crystallinity of Silk Fibers

**Fig. 3:**
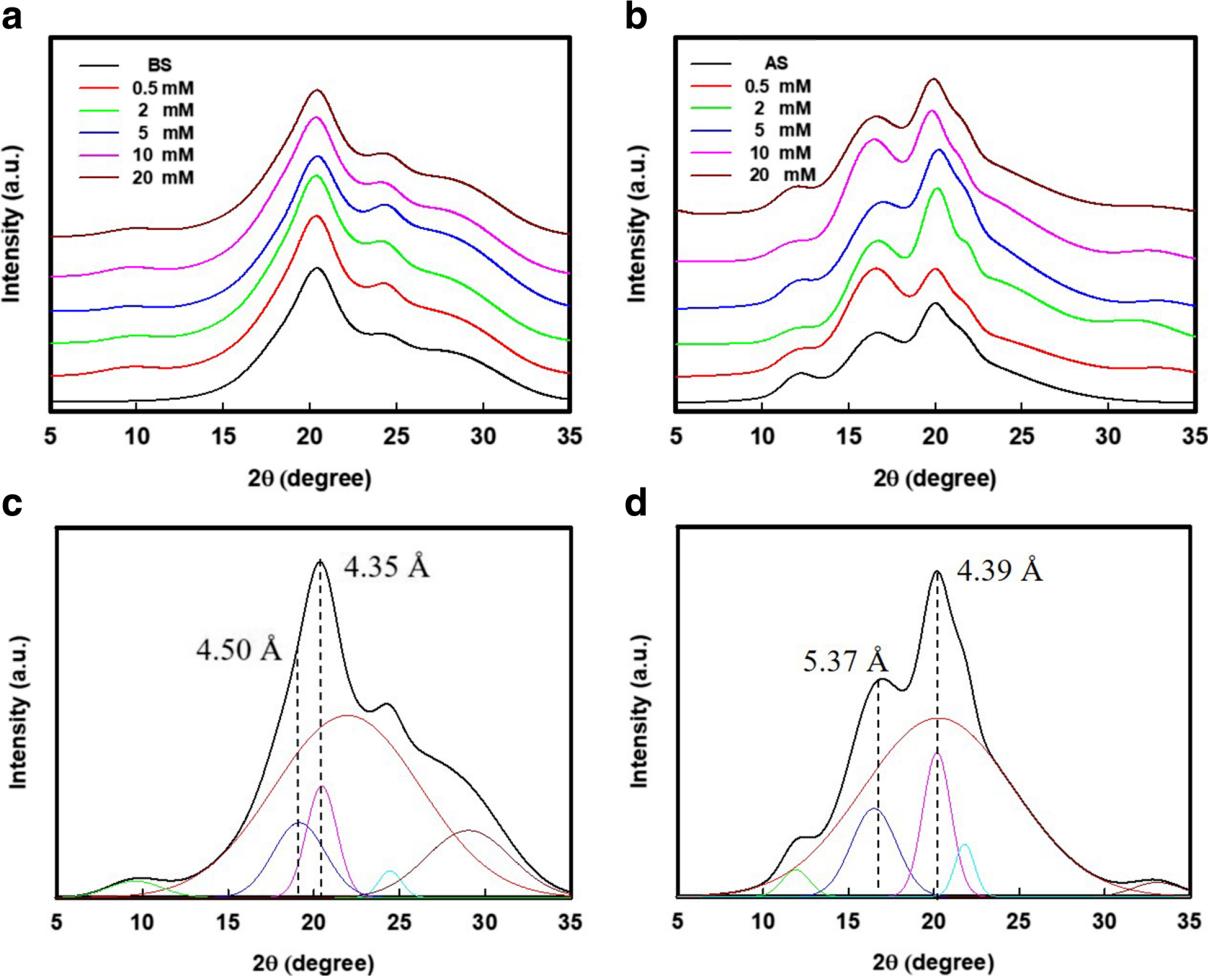
XRD analysis of the oxidized silk fibers. X-ray diffraction (XRD) pattern of **a** BS and **b** AS that oxidized with various NaClO addition. Representative deconvolution and results of **c** BS and **d** AS materials

The silk protein molecules acted as hydrophilic-hydrophobic-hydrophilic polymers, which folded into irregular-sized micelles during the formation of hydrophilic beads (amorphous regions) extending out from hydrophobic cores (crystalline regions) [17]. The SNs were assembled due to the adhesion of the outer regions between the micelles. However, it is suggested that the NaClO oxidation of silk fibers proposed a weak adhesion between their nanostructures [25]. As shown in Fig. 3a and b, after oxidation, the X-ray diffraction (XRD) patterns of oxidized BS fibers were similar to their original pattern, as well as the XRD patterns of oxidized AS fibers. Thus, the oxidized silk fibers remained their natural nano-building block, i.e., β-sheet structures in silk fibers. On the other hand, the deconvolution of these XRD patterns (Fig. 3c, d) suggested a significant change of crystallinity in both BS and AS fibers after oxidation, where the details were listed in Table 1. Although oxidation mainly occurred on the serine residues of the silk protein, there were several amino groups in the amorphous regions that could be attacked by NaClO [29]. Therefore, it is understandable that the crystallinity of the oxidized BS fibers in Table 1 increased from 24.8% (disassembled BS) to 41.3% (with the addition of 10 mM/g of protein NaClO), followed by an increase in the carboxyl content. A similar tendency was also presented in the case of oxidized AS fibers, where the crystallinity of these AS fibers was increased from 22.9 to 39.2%. The results suggest that, besides the electrostatic repulsion forces, the destruction of amorphous regions in the silk proteins was also an important factor in dispersing the SNs. The crystallinity of the oxidized silk fibers (both BS and AS) was followed by the increasing carboxyl content when the NaClO addition was < 10 mM/g of protein. The degradation of the amorphous regions is prior to the crystallized cores of the silk protein. However, excess amounts of NaClO (20 mM/g of protein) may possibly degrade the silk. This phenomenon is in good agreement with the results that we revealed in Fig. 1b and c.

**Table 1:** The crystallinity and carboxyl content of BS and AS pulp after oxidation with various NaClO addition

NaClO added (mM/g protein)		N/A	0.5	2	5	10	20
BS	Carboxyl content (mM/g protein)	0.297	0.381	0.511	0.592	0.720	0.899
	Crystallinity (%)	24.8	26.8	28.9	38.4	41.3	35.2
AS	Carboxyl content (mM/g protein)	0.343	0.434	0.492	0.665	0.833	1.01
	Crystallinity (%)	22.9	23.1	32.9	38.1	39.2	33.3

### The Performance of Silk Nanofibers

**Fig. 4:**
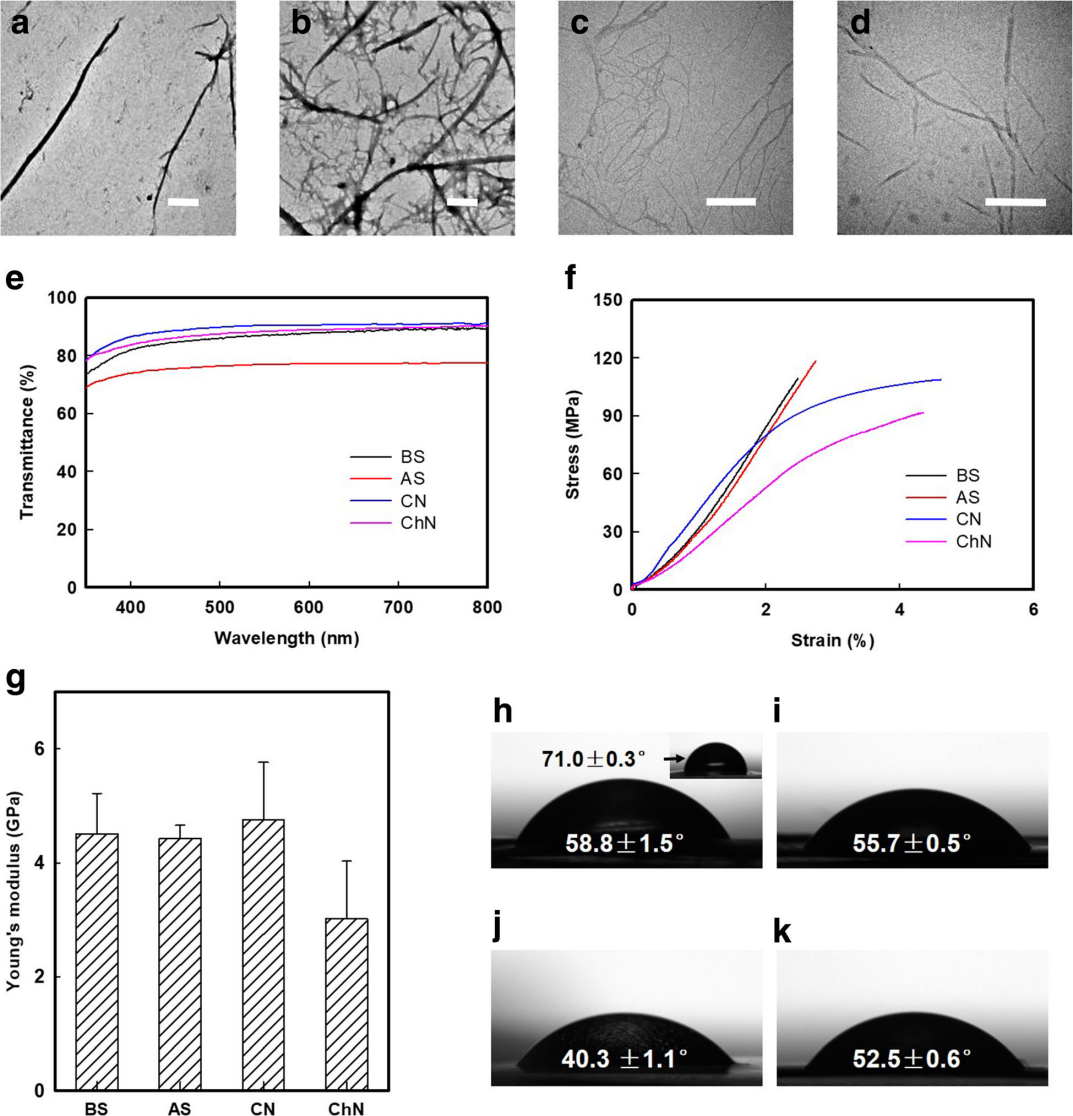
Morphology and properties testing of SNs, CNs, and ChNs. Transmission electron microscopy (TEM) observation of resultant **a** BS and **b** AS nanofibers that oxidized by 10 mM/g protein NaClO addition, **c** cellulose nanofibers (CNs), and **d** chitin nanofibers (ChNs) that achieved by TEMPO-mediated oxidation. The scale bar is 500 nm. **e** UV-Vis transmittance of approximately 50-μm-thick membranes that cast by BS, AS, cellulose (CN), and chitin (ChN) nanofibers. **f** Representative stress-strain curves of approximately 50-μm-thick membranes that cast by BS, AS, CN, and ChN nanofibers. **g** Young’s modulus of membranes that are casting from BS, AS, CN, and ChN nanofibers. Data represent the mean SD (*n* = 5). **h**–**k** The water contact angle of membrane cast by **f** BS nanofibers was 58.8 ± 1.5°, significantly reduced from that of regenerated BS membrane (71.0 ± 0.3°, the inset image). 55.7 ± 0.5, 40.3 ± 1.1, and 52.5 ± 0.6° of water contact angle was presented in AS, CN, and ChN membrane, respectively

The morphologies of the oxidized BS and AS nanofibers that were obtained by ultrasonication of 10 mM NaClO oxidized silk fibers are shown in Fig. 4a and b. The BS and AS nanofibers have a similar aspect ratio (calculated by the ImageJ software), where 16.92 for BS nanofibers on average and 19.12 for AS nanofibers, respectively. In comparison, the cellulose nanofibers (CNs) and chitin nanofibers (ChNs) prepared using TEMPO-mediated oxidation are shown in Figs. 4c and d. To further characterize these SNs, approximately 50-μm-thick membranes were cast by using a solvent evaporation method. Optically transparent (above 75% transmission) silk membranes were evaluated using a UV-Vis (from 350 to 800 nm) spectrophotometer (Fig. 4e).

The nanofibers obtained from this downsizing method retained a highly crystalline structure and high aspect ratio. As a result, these membranes presented robust mechanical properties (Fig. 4g) with Young’s moduli of 4.51 ± 0.71 and 4.43 ± 0.23 GPa for BS and AS, respectively, which were comparable to those of the CN and ChN membranes (the representative strain and stress curves are given in Fig. 4f). Furthermore, the wetting properties of the BS membrane were significantly improved in the regenerated membrane due to the introduction of carboxyl groups. As shown in Fig. 4h, the water contact angle of BS nanofibers casting membrane is 58.8 ± 1.5°, while the regenerated BS membrane (the inset image in Fig. 4h) is 71.0 ± 0.3°. In addition, 55.7 ± 0.5, 40.3 ± 1.1 and 52.5 ± 0.6° of water contact angle was presented in AS (Fig. 4i), CN (Fig. 4j), and ChN (Fig. 4k) membrane, respectively.

**Fig. 5:**
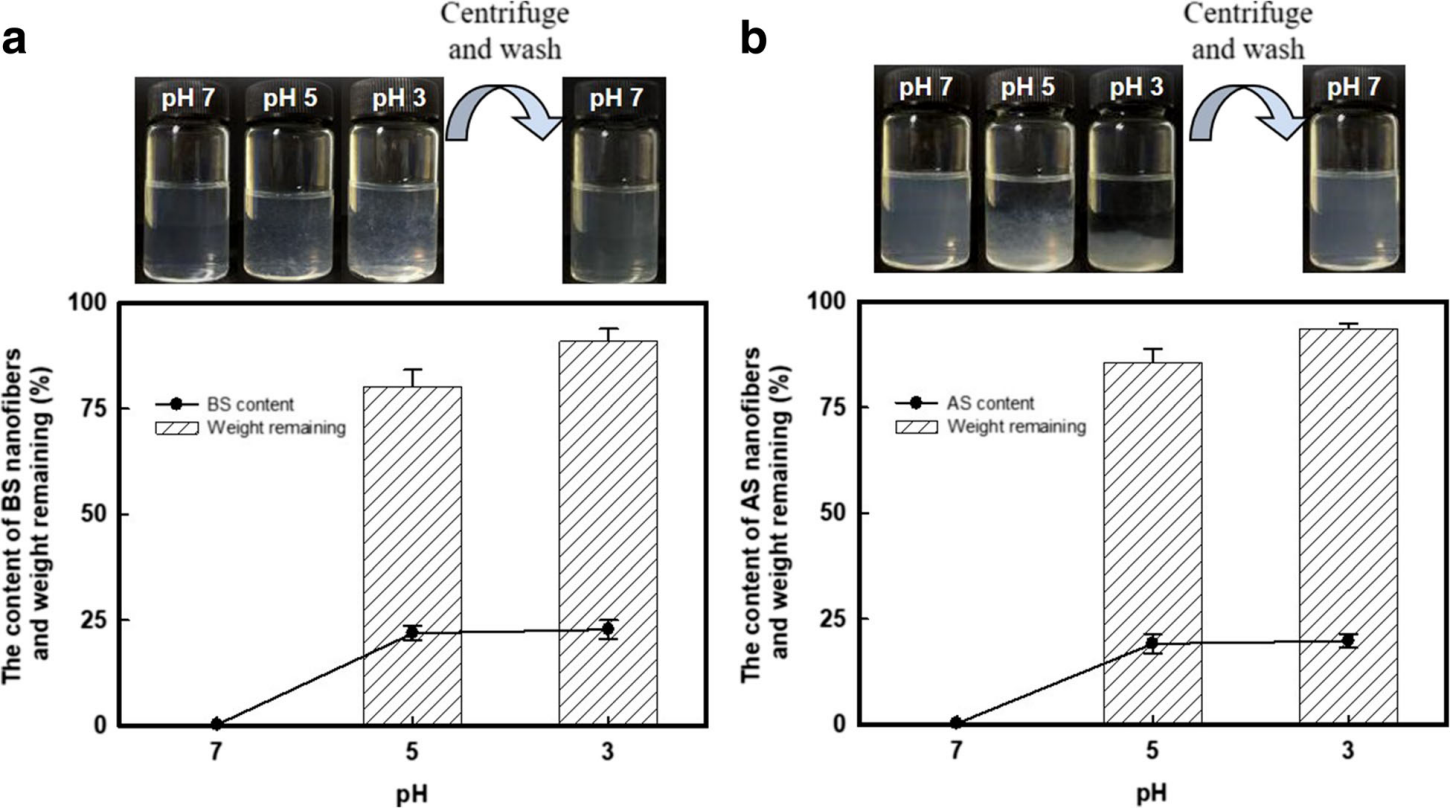
Re-dispersing process of SNs. Photography of the pH response phenomenon for **a** BS and **b** AS nanofibers. Over 80 wt% of the proteins (both BS and AS) were remaining after centrifugation, with the protein content of ~ 20 wt%

Both CN and ChN and silk devices have been extensively applied in materials science for decades [13, 30, 31], due to their similar mechanically robustness, processing plasticity and biochemical properties, etc. Of course, intrinsic differences exist in these polysaccharide- and protein-based materials. We therefore wondered how their differences regulated the nanofiber formation. The properly dispersed BS and AS dispersions had a zeta-potential of − 39.5 ± 0.66 and − 37.4 ± 2.4 mV, respectively, under neutral conditions. The electrostatic repulsions between carboxyl groups are against the adhesion between silk micro-/nano-fibril interfaces; thus, these nanofibers dispersed in aqueous phase homogeneously. Interestingly, when the pH decreased, the H^+^ shielded the negatively charged surfaces leading to aggregation of the nanofibers, as shown in Fig. 5a and b. The aggregates of the SNs could be re-dispersed in water by adjusting the pH > 7, or they could be easily collected after centrifugation and then re-dispersing with slightly stirring. The bottom charts of Fig. 5 show the remaining weight of the SN aggregates collected under different pH conditions. For the BS, 80.1 ± 1.7 and 90.9 ± 2.2 wt% (85.7 ± 2.2 and 93.6 ± 1.5 wt% for AS) of the aggregates was recovered at pH 5 and 3, respectively. Meanwhile, this process concentrated the SNs by approximately 100 times (~ 20 wt%) compared to the initial dispersion, with a concentration of ~ 0.2 wt%. This fascinating property of the SNs was attributed to (i) the intrinsic pH response of the protein-based materials and (ii) the flexibility of the soft matter SNs during the aggregation and re-dispersion processes. The aggregation-redispersion phenomenon suggested a promising application of these SNs as drug loading and releasing carriers. In addition, there has been no dispute that the resultant SNs are well appropriate for storage and transportation.

## Conclusions

In summary, individual dispersed BS and AS nanofibers were achieved after NaClO oxidation. The approach was similar to the TEMPO-mediated oxidation of polysaccharides to prepare nanofibers; however, TEMPO/NaBr catalysts were not required. The as-prepared SNs were ~ 110 nm in diameter and several microns long, with negatively charged surfaces. Optically transparent, mechanically robust, and enhanced wetting properties were obtained in the SN membranes. In particular, the SNs could be concentrated to ~ 20 wt% by lowering the pH, and these pulp-like SNs were re-dispersible in neutral aqueous solutions. Based on these results, the SNs are an excellent candidate for material science and biomedical applications.

## Additional file

Additional file 1: The Raman spectra of raw BS fibers and formic acid pretreated BS fibers. (DOCX 51 kb)

## Data Availability

All data generated or analyzed during this study are included in this published article.

## Abbreviations

AS: *Antheraea pernyi* silk
BS: *Bombyx mori* silk
ChN: Chitin nanofiber
CN: Cellulose nanofiber
SEM: Scanning electron microscopy
SN: Silk nanofiber
TEM: Transmission electron microscopy
XRD: X-ray diffraction
