# Supplementary material for: Oxidizing and Nano-dispersing the Natural Silk Fibers
Source: Nanoscale Res Lett. 2019 Jul 25;14:250. doi: 10.1186/s11671-019-3080-1 (PMC6658644; doi:10.1186/s11671-019-3080-1)
Supplement: Supplementary file 1 — The Raman spectra of raw BS fibers and formic acid pretreated BS fibers. (DOCX 51 kb) [file 11671_2019_3080_MOESM1_ESM.docx]

Oxidizing and Nano-Dispersing the Natural Silk Fibers

Ke Zheng ^1,2^, Yanlei Hu^1^, Wenwen Zhang ^1,2^, Juan Yu ^1^, Shengjie Ling ^2,^* and Yimin Fan ^1,^*

^1^ Jiangsu Co-Innovation Center of Efficient Processing and Utilization of Forest Resources, Jiangsu Key Lab of Biomass-based Green Fuel & Chemicals, Key Laboratory of Forestry Genetics & Biotechnology (Nanjing Forestry University) of Ministry of Education, College of Chemical Engineering, Nanjing Forestry University, Nanjing 210037, China

^2^ School of Physical Science and Technology, ShanghaiTech University, Shanghai, 201210, China

*Corresponding author

Tex./fax: Y. Fan: +86 025 85427215

Email address: Y. Fan: [fanyimin@njfu.edu.cn](mailto:fanyimin@njfu.edu.cn); S. Ling: [lingshj@shanghaitech.edu.cn](mailto:lingshj@shanghaitech.edu.cn)

**Supporting Information**

Figure S1, The Raman spectra of raw BS fibers and formic acid pretreated BS fibers.

It is important to investigate the possible chemical reaction between amino acid or hydroxyl groups with formic acid or NaClO. Here, we further investigated the possible chemical reaction between silk fibers and formic acid by Raman spectra. The Raman spectra of raw BS fibers and pretreated BS fibers is shown below, and suggested that there is no significantly chemical change of BS fibers after the formic acid treatment. It is understandable because the formic acid pretreatment process is only employed to disassemble the silk fibers.

**Figure S1.** The Raman spectra of raw BS fibers and formic acid pretreated BS fibers.

In addition, reactions between NaClO and proteins have been widely studied [Proteins: Struct., Funct., Bioinf. 2001, 44, 119-122; Amino Acids 2003, 25, 259-274.]. The amino acid composition of silk fibroin consists primarily (over 85% present of silk’s total amino acid sequences) of glycine, alanine and serine. As non-reactive amino acids, glycine (-H) and alanine (-CH3), no residues for reaction are present. The chemical reaction between hydroxyl groups and NaClO (i.e. carboxyl content) was investigated in our manuscript. The extensive tyrosine hydroxyl groups (~ 5% of the amino acids per silk fibroin chain) were excluded here because the main reaction with NaClO and tyrosine is chlorination with the aromatic ring [Amino Acids 2003, 25, 259-274]. Otherwise, other amino groups in the SF protein that may be attacked by NaClO as well, but the contribution here is negligible due to the minor abundance (< 1%) [Proteins: Struct., Funct., Bioinf. 2001, 44, 119-122]. Therefore, we are focused to characterize the oxidation between the hydroxyl groups and NaClO.
